# R4ST: a reference-guided graph-generative model for robust reconstruction of spatial transcriptomic profiles

**DOI:** 10.1093/bioinformatics/btag228

**Published:** 2026-07-07

**Authors:** Mingyue Wei, Wenrui Li, Wei Zhang, Zhi-Ping Liu

**Affiliations:** Department of Biomedical Engineering, School of Control Science and Engineering, Shandong University, Jinan, Shandong 250061, China; Department of Automation, Tsinghua University, Beijing 100084, China; Department of Biomedical Engineering, School of Control Science and Engineering, Shandong University, Jinan, Shandong 250061, China; Department of Biomedical Engineering, School of Control Science and Engineering, Shandong University, Jinan, Shandong 250061, China

## Abstract

**Motivation:**

The trade-off between spatial granularity and transcriptome coverage in current spatial transcriptomics (ST) technologies results in sparse and incomplete expression profiles. Meanwhile, the rich local and global spatial topology inherent in spatial data are crucial for accurate biological interpretation but remain underutilized by existing methods.

**Results:**

Here, we propose R4ST, an end-to-end framework designed to complete ST data. R4ST leverages scRNA-seq data as a reference and employs dual learning channels based on graph inductive and transductive modeling to capture complementary spatial topology information in ST data, enabling accurate reconstruction of missing gene expression. Extensive evaluations across multiple datasets from different platforms demonstrate that R4ST enables accurate recovery of large-scale gene expression profiles from a small subset of measured genes, uncovers novel spatial patterns associated with rare cell types, and substantially enhances the biological interpretability of ST data.

**Availability:**

https://github.com/zpliulab/R4ST

## 1 Introduction

In recent years, spatial transcriptomics (ST) technologies have enabled gene expression measurement within intact tissue sections while preserving spatial context, extending transcriptomic profiling from the cellular to the spatial dimension (Zhang *et al.* 2024). This advancement has provided unprecedented opportunities to investigate tissue architecture, cellular interactions, and spatial mechanisms underlying tissue development and disease progression.

ST technologies can be broadly categorized into image-based and sequence-based approaches, each offering distinct advantages in spatial resolution, throughput, and experimental design (Sun *et al.* 2025). Image-based methods typically achieve single-cell or subcellular resolution, while sequencing-based platforms enable transcriptome-wide profiling across larger tissue areas. Despite these advances, ST data remain constrained by inherent technical limitations, particularly signal sparsity and limited gene coverage (Li *et al.* 2025). High spatial resolution often comes at the expense of reduced gene detection sensitivity and fewer genes measured per spatial unit, resulting in sparse expression matrices and incomplete transcriptomic profiles. This trade-off between spatial granularity and transcriptome coverage poses significant challenges for downstream analyses and limits the comprehensive interpretation of spatial gene expression patterns.

Over the past few years, several methods for ST data completion have been proposed, leveraging different mechanisms to jointly model spatial context and gene expression. For example, embedding-based alignment methods, such as SpaGE (Abdelaal *et al.* 2020) and stPlus (Shengquan *et al.* 2021), impute missing genes through local averaging in a low-dimensional embedded space. However, these approaches either implicitly model spatial relationships or introduce them as a post-processing step, weakening spatial information modeling and leading to blurred spatial structure identification. Deep generative and encoder-decoder models, including stDiff (Li *et al.* 2024) and stAI (Zou *et al.* 2025), use neural networks to learn complex non-linear mappings but do not treat cross-modality topological structure as a primary modeling component. Additionally, methods like novoSpaRc (Moriel *et al.* 2021) and SpaOTsc (Cang and Nie 2020) employ optimal transport algorithms to map scRNA-seq to ST data, but their high computational complexity limits scalability to ultra-large ST datasets. Improved frameworks such as SPRITE (Sun *et al.* 2024) and TISSUE (Sun et al. 2024) enhance foundational methods but remain heavily dependent on the quality of original spatial data, occasionally resulting in suboptimal outcomes or noise propagation (Yang and Zhang 2023).

To address these challenges, we developed R4ST, a method specifically designed for ST data completion. R4ST uses a shared latent space between scRNA-seq and ST data as an anchor, avoiding direct generation in high-dimensional spaces and reducing the risk of out-of-distribution extrapolation during imputation. R4ST is mainly intended for image-based ST data, which, though providing subcellular spatial resolution, are often constrained by limited gene coverage, impeding thorough downstream analyses. Unlike existing approaches, R4ST constructs dual learning channels with distinct semantic roles: an inductive channel learns transferable neighborhood aggregation patterns, while a transductive channel leverages the full spatial graph structure to enforce global structural consistency. Additionally, R4ST explicitly models spatial uncertainty through variational graph encoders, enabling uncertainty-aware gene imputation grounded in spatial topology. Experiments on both image-based and sequence-based datasets demonstrate that R4ST achieves accurate and spatially consistent gene imputation while preserving key expression and spatial patterns critical for downstream analyses.

## 2 Materials and methods

### 2.1 R4ST algorithm

**Figure 1:**
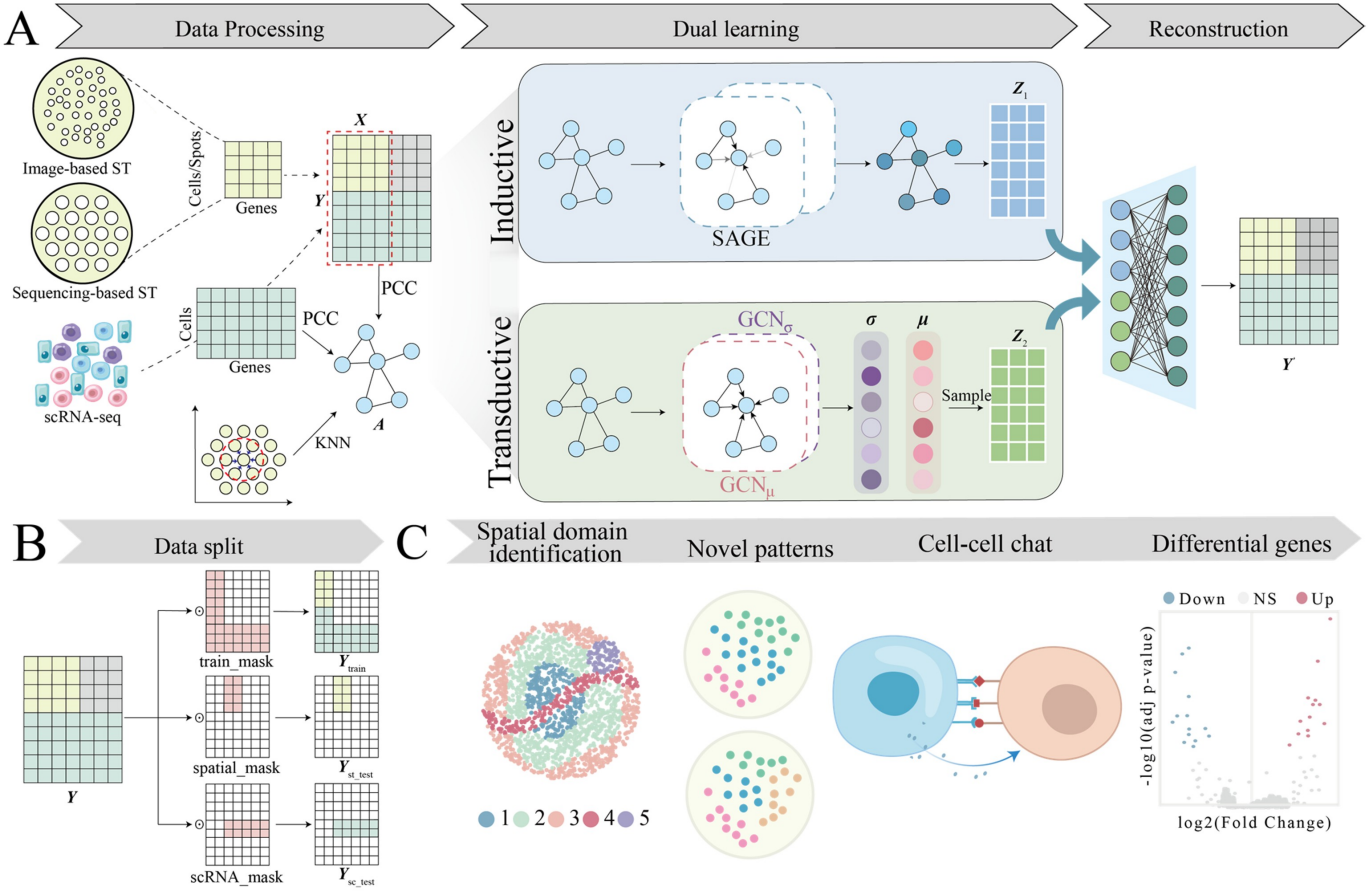
R4ST framework for spatial transcriptomics imputation and downstream analysis. (A) Data processing and model architecture. (B) Data splitting strategy. (C) Downstream applications.

The flowchart of R4ST is shown in Fig. 1. Its core architecture is based on a variational graph autoencoder (VGAE) (Kipf and Welling 2016), utilizing cellular neighborhood topology to constrain latent space for systematic ST data reconstruction, enhancing cellular feature representations and providing a robust basis for downstream, cell-centric biological analyses. R4ST uses paired scRNA-seq data as a reference. Highly variable genes not shared between scRNA-seq and ST data are selected as prediction targets. To study biologically important but non-highly variable genes like transcription factors, the framework permits manual supplementation of the target gene list for fine-grained biological exploration. For genes detected in scRNA-seq but not detected in ST, their expression values at corresponding spatial locations are set to zero. The scRNA-seq and ST data are concatenated to form a gene expression matrix Y, where the upper part corresponds to the ST data and the lower part corresponds to single-cell data. The zero-valued region in the upper-right corner represents missing genes in ST data and is the reconstruction target.

Let $X_{\mathrm{st}}\in \;R^{m\times p}$ denote ST data with m spots and p genes, and $X_{\mathrm{sc}}\in \;R^{n\times q}$ denote reference scRNA-seq data with n cells and q genes. R4ST selects the D genes shared by the ST and scRNA-seq datasets as the input feature matrix $X\in \;R^{N\times D}$, where N=m+n represents the total number of cells. A full adjacency matrix $A\in \;R^{N\times N}$ is built to capture complex cellular relationships. As cells in the same spatial area often share similar cell types or morphological states, we initially use the KNN algorithm on spatial coordinates from ST data to capture local tissue microenvironments. However, in complex tissue samples, the same cell types may be spatially separated. To address this, we also create edges based on transcriptional similarity. A strict threshold of 0.9 is applied to scRNA-seq data for high-confidence interactions, while a relaxed threshold of 0.5 is used for cross-modal integration to reduce batch effects and retain broader biological signals. These adjacency relationships are integrated to form the overall adjacency matrix A. Finally, the feature matrix X and the adjacency matrix A are subsequently fed into R4ST to reconstruct the gene expression matrix $Y\in \;R^{N\times Q}$, where Q represents the union of shared genes and highly variable genes (HVGs). The main goal is to predict the missing expression values for the highly variable genes within the ST component of this matrix.

Many existing methods model spatial topological relationships of ST data either implicitly or introduce spatial information only as a post-processing step, making it hard to fully capture complex topological structures in ST data. To address this, a dual learning channel strategy is proposed (Fig. 1A).

In the graph inductive learning channel, R4ST builds a neighborhood aggregation model based on GraphSAGE (Hamilton *et al.* 2018) to learn local spatial neighborhood aggregation patterns in ST data. This channel has two layers of GraphSAGE. By sampling and aggregating features from neighboring cells, it reduces noise impact and enhances robustness to local structural perturbations, stably characterizing expression relationships within spatial neighborhoods and generating latent representation $Z_{1}$. For a cell v and its neighbors N(v), the aggregation and update process for layer k is:


$$h_{N(v)}^{k}={\mathrm{AGGREGATE}}_{k}(\left\{h_{u}^{\left(k-1\right)},\forall u\in N(v)\right\}),$$

$$h_{v}^{\left(k\right)}=\sigma (W^{\left(k\right)}\cdot \mathrm{CONCAT}(h_{v}^{\left(k-1\right)},h_{N(v)}^{k})).$$

where $h_{v}^{k}$ is the feature vector of cell v at layer k, $W^{\left(k\right)}$ is the learnable weight matrix for layer k. The initial input is $h_{v}^{0}=X_{v}$.

Since R4ST uses two layers of GraphSAGE, the final latent representation $Z_{1}$ is:


$$Z_{1}=h_{v}^{\left(2\right)}$$

In the graph transductive learning channel, R4ST employs a Graph Convolutional Network (GCN) (Gilmer *et al.* 2017) to model the complete spatial graph, explicitly leveraging the global spatial topology inherent in ST data. Within the VGAE framework, this channel learns the mean μ and variance σ of the latent representations and obtains latent representation $Z_{2}$ through variational sampling.


$$\mu ={\mathrm{GCN}}_{\mu }(X,A)=\tilde{A}\mathrm{Relu}(\tilde{A}XW_{0})W_{\mu },$$

$$\log\sigma ={\mathrm{GCN}}_{\sigma }(X,A)=\tilde{A}\mathrm{Relu}(\tilde{A}XW_{0})W_{\sigma },$$

$$Z_{2}=\;\mu +\sigma ⊙\varepsilon ,\;\varepsilon \in Ν(0,I).$$

where the $W_{0},\;W_{\mu },\;$and $W_{\sigma }$ are trainable weights. The normalized adjacency matrix is defined as $\tilde{A}=D^{-\frac{1}{2}}AD^{-\frac{1}{2}}$; here, D is the diagonal degree matrix with $D_{\mathrm{ii}}=\sum_{j=1}^{N}A_{\mathrm{ij}}$, and ⊙ denotes the Hadamard product.

After obtaining $Z_{2}$, the merged latent representation **Z** is obtained by concatenating $Z_{2}$ and $Z_{1}$, and the reconstructed adjacency matrix $\hat{A}$ is generated as:


$$\hat{A}=\mathrm{sigmoid}(Z\cdot Z^{T})$$

By explicitly modeling spatial uncertainty with the variational graph encoder, R4ST enables uncertainty-aware gene expression imputation under spatial topological constraints, improving the consistency and reliability of reconstruction results with respect to the global spatial structure.

A subset of shared gene expression data between ST and scRNA-seq is retained for model training and evaluation. Specifically, train_mask, spatial_mask, and scRNA_mask are used as mask matrices to split the gene expression matrix Y into training, spatial transcriptomics test, and scRNA-seq test sets (Fig. 1).


$$Y_{\mathrm{train}}=\mathrm{train}\_\mathrm{mask}⊙Y,$$

$$Y_{\mathrm{st}\_\mathrm{test}}=\mathrm{spatial}\_\mathrm{mask}⊙Y,$$

$$Y_{\mathrm{sc}\_\mathrm{test}}=\mathrm{scRNA}\_\mathrm{mask}⊙Y.$$

Here, train_mask, spatial_mask, and scRNA_mask$\;\in {\left\{0,1\right\}}^{N\times Q}\;$are binary matrices, with elements corresponding to the training, spatial transcriptomics test, and scRNA-seq test sets set to 1. The values in $Y_{\mathrm{train}}\;$are used as ground truth to compute the mean squared error between the true values and model predictions $Y^{\prime }$, yielding the training feature loss (${\mathrm{loss}}_{\mathrm{feature}}$). The model is optimized through a total loss function:


$${\mathrm{Loss}}_{\mathrm{total}}={\mathrm{loss}}_{\mathrm{feature}}+\alpha \cdot \mathrm{los}s_{\mathrm{graph}}+\beta \cdot \mathrm{los}s_{\mathrm{kl}},$$

where α, β are weighting hyperparameter. Specifically, the training feature loss is calculated as ${\mathrm{loss}}_{\mathrm{feature}}=\;\mathrm{MSE}(Y_{\mathrm{train}},\;Y^{\prime })$. To preserve the global spatial topology, the graph reconstruction loss is defined as $l_{\mathrm{graph}}=\mathrm{CrossEntropy}(A,\;\hat{A})$. Finally, $\mathrm{los}s_{\mathrm{kl}}$ denotes the Kullback-Leibler (KL) divergence, defined as ${\mathrm{los}s_{\mathrm{kl}}=D}_{\mathrm{KL}}(q(Z|X,A)||N(0,I))$, which regularizes the latent distribution q(Z|X,A) to align with a standard normal distribution.

### 2.2 Datasets and benchmarking methods

To comprehensively evaluate the performance of R4ST, we used five pairs of spatial transcriptomics (ST) and scRNA-seq datasets. These datasets cover various ST modalities, divided into: (i) image-based methods, such as MERFISH, seqFISH, and STARmap; and (ii) sequencing-based methods, specifically Slide-seq V2. This ensures a robust assessment across platforms with different spatial resolutions and gene throughputs.

To rigorously test the imputation performance of R4ST, we adopted a systematic masking-and-imputation approach. For each dataset, we first identified gene common to both ST data and scRNA-seq reference. These common genes were then split into two distinct subsets: input features for model training and held-out target genes for quantitative benchmarking. Detailed statistics for each dataset, including cell counts, total common genes, and input features used, are provided in Table S1.

We compared R4ST with five methods: SPRITE (a meta-algorithm for prediction refinement), stAI (an encoder-decoder model for joint embedding), stDiff (a latent diffusion generative approach), novoSpaRc (an optimal transport-based mapping method), and SpaGE (an SVD-based statistical projection method), which are widely used and have demonstrated strong performance (Li *et al.* 2022). Multiple metrics were used for evaluation, including PCC, SCC, Tau, MNI, ARI, Homo, completeness, V-measure, and FMI. See Supplementary Section 1 for details.

### 2.3 Implementation and hyperparameters

We employed the Scanpy package to data preprocessing, including filtering low-quality genes and cells, followed by total-count normalization and log(1+x) transformation to ensure cross-platform data comparability. Data sources and additional details are provided in Table S1 and Section 3. The model was trained using a learning rate of $1\times 10^{-5}$, with loss weights α=0.001 and β=0.1, and an early stopping patience of 10. These hyperparameters were selected through grid search on validation sets and kept consistent across datasets, except for patience, which was adjusted as specified in the Supplementary Section 2.

## 3 Results

### 3.1 Benchmarking and ablation study

Our assessment of R4ST across five diverse spatial transcriptomics datasets revealed consistently superior performance compared to existing baselines. As summarized in Table S3, R4ST demonstrates strong predictive accuracy and structural preservation across all benchmarks. Quantitatively, R4ST achieves an average improvement of 0.071 in median PCC across datasets. Compared to the suboptimal methods, R4ST achieved a maximum improvement of 40% in the median PCC value. While correlation values differ across datasets, these variations stem from platform-specific differences in sparsity and technical noise. Importantly, R4ST maintains notable performance advantages in high-sparsity settings, such as the seqFISH dataset, where other methods fail to capture valid correlations. Beyond numerical recovery, R4ST consistently scores higher in clustering metrics, confirming its ability to preserve latent spatial structures for reliable downstream biological analysis.

Additionally, ablation studies confirm that the dual-channel module substantially improves correlation coefficients and reduces reconstruction errors compared to the transductive baseline. These improvements validate the effectiveness of our dual-channel architecture in optimizing local expression logic for the recovery of highly variable genes (Fig. S1).

### 3.2 R4ST enables spatial gene imputation from sparse markers and reveals novel spatial patterns in the mouse brain

Identifying spatial domains from ST data is crucial for understanding tissue organization and functional heterogeneity. MERFISH is a ST technology that enables in situ quantification by combining combinatorial barcoding with fluorescence imaging rounds, achieving cell-level resolution (Xia *et al.* 2019). However, due to experimental design and imaging throughput limitations, MERFISH typically detects only hundreds to thousands of RNA species, resulting in limited gene coverage compared to sequencing-based methods.

**Figure 2:**
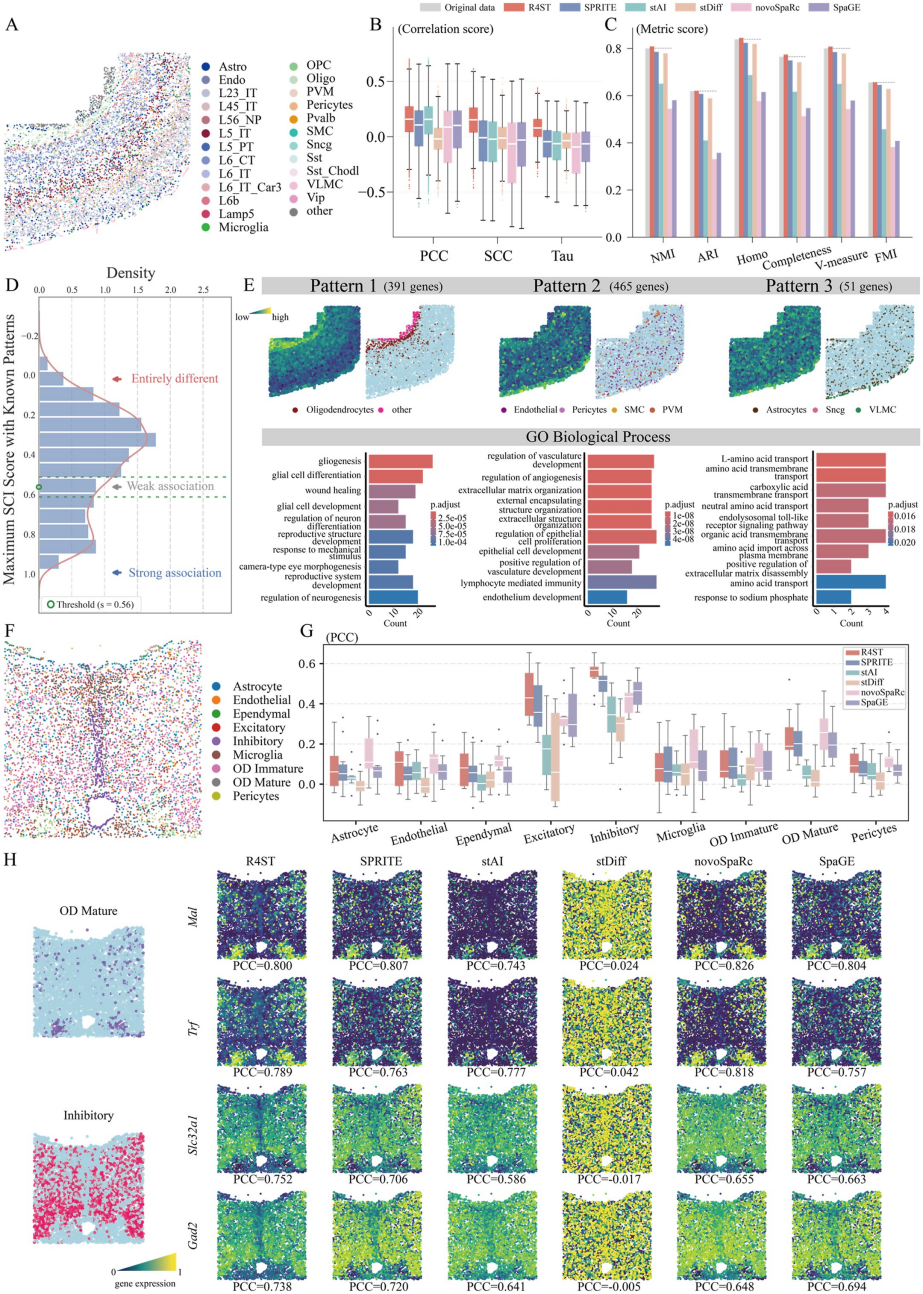
Performance evaluation of R4ST and competing methods on MERFISH datasets. (A) Spatial distribution of original cell-type labels. (B) Box plots of coefficients for different methods. (C) Clustering consistency evaluation of imputed data across six metrics. (D) Distribution of likelihood scores used for gene categorization. Unmeasured genes are divided into three confidence groups by two dashed lines. The green point denotes the local minimum between the likelihood peaks, with dashed lines positioned at s ± 0.05. (E) Spatial patterns of three newly identified patterns, with associated cell types and GO terms. (F) Original cell-type labels in mouse preoptic area of hypothalamus. (G) The PCC boxplot for top 10 marker genes across cell types. (H) Spatial visualization of predicted expression for unmeasured genes.

To evaluate R4ST’s performance in addressing gene incompleteness and identifying spatial domains in ST data, we tested a MERFISH dataset from the mouse primary motor cortex (Booeshaghi *et al.* 2021) (Fig. 2A). This dataset contains 254 genes and 6963 cells. A 10× Chromium dataset was used as the paired reference (Booeshaghi *et al.* 2021). We selected the 200 genes shared between ST and scRNA-seq datasets as input features. R4ST was used to impute the expression values of remaining 52 measured common genes and 2000 highly variable genes (HVGs) not measured in ST data but variable in scRNA-seq data.

We first assessed R4ST and other methods in gene expression prediction and cell clustering consistency (Fig. 2B and C). R4ST accurately predicted unmeasured gene expression levels in ST data, achieving higher correlations between predicted and true expression values than competing methods (Fig. 2B). This is attributed to R4ST’s inductive channel based on GraphSAGE, which learns transferable neighborhood aggregation patterns from scRNA-seq reference. For cell clustering, R4ST performed best across all six metrics, indicating its ability to learn and integrate gene expression patterns from different cell types, improving accuracy and robustness. Notably, when comparing R4ST-imputed data with ground-truth labels, clustering consistency was only slightly lower than original ST data in terms of FMI metric, while outperforming original data on the remaining five metrics. This suggests that by incorporating reference information and learning the underlying expression structure, R4ST can mitigate noise and systematic errors in MERFISH, enhancing original ST data and reducing the impact of technical artifacts on spatial domain identification.

To evaluate the biological relevance of R4ST-imputed genes, we applied the same spatial pattern identification pipeline to original and imputed ST data. This comparison focused on whether imputed data could reveal additional spatial patterns not identifiable from original data. Using original ST data, we employed MERINGUE (Miller *et al.* 2021) to characterize spatial organization of measured genes. A weighted gene co-expression network was constructed by computing spatial cross-correlation index (SCI) between gene pairs, followed by network clustering. This analysis identified five spatial expression patterns co-localized with known cell types (Fig. S2), treated as reference spatial patterns supported by original data. We then used the imputed ST data to assess whether newly predicted genes introduced spatial structural information beyond original patterns. For imputed data, we computed likelihood scores between predicted genes and five patterns identified from original data, quantifying spatial similarity and deviation (Fig. 2D). Based on these scores, missing genes were classified into three categories: (i) genes strongly associated with reference patterns, (ii) genes weakly associated with reference patterns, and (iii) genes that significantly deviated from all reference patterns. Genes in the third category were considered most likely to correspond to novel spatial structures. we further applied MERINGUE to cluster spatial expression profiles of these predicted genes that markedly deviated from reference patterns. As a result, imputed ST data enabled identification of three additional spatial patterns distinct from each other and clearly separated from original five reference patterns (Fig. 2E).

To validate the biological plausibility and functional relevance of newly identified spatial patterns, we conducted a systematic analysis from two perspectives: cell-type co-localization and functional enrichment analysis. Results showed that pattern 1 was primarily co-localized with oligodendrocytes and several other cell types (Fig. 2E). Oligodendrocytes play a critical role in the central nervous system by forming myelin sheaths that support and insulate axons (Sherman and Brophy 2005). Functional enrichment analysis revealed that genes associated with this pattern were significantly enriched in biological processes related to oligodendrocytes, including gliogenesis and glial cell differentiation. Additionally, gene ontology (GO) enrichment results indicated that this pattern was also involved in nervous system and visual system development, as well as responses to mechanical stimuli, suggesting it may reflect functional characteristics of multiple cell types in the mouse primary motor cortex. Pattern 2 exhibited co-localization with multiple vascular-associated cell types, including endothelial cells, perivascular macrophages (PVM), smooth muscle cells (SMCs) and pericytes. These cell types collectively contribute to vascular structure maintenance and blood circulation regulation (Dudley and Griffioen 2023). Consistently, functional enrichment analysis showed that genes in this pattern were mainly involved in biological processes related to vascular development and regulation, tissue remodeling, and epithelial development. Pattern 3 was predominantly co-localized with astrocytes. Astrocytes are a central cell type in the brain and are known for their diverse functions, including maintenance of the blood–brain barrier, support of neural development and repair, and provision of metabolic support (Ullian 2012). Functional enrichment analysis indicated that genes in this pattern were significantly enriched in a wide range of biological processes, including immune response, metabolic regulation, and signal transduction, further supporting the functional specificity of this spatial pattern.

Further, we systematically evaluated the performance of R4ST in imputing missing marker genes across different cell types using the mouse hypothalamus MERFISH dataset (Fig. 2F). Given the critical role of cell type–specific marker genes in downstream analyses, such as cell type annotation and functional characterization, we focused on assessing the predictive accuracy of R4ST for these genes. For each cell type, the top 10 representative marker genes were selected (Supplementary Section 2.3), and imputation performance was quantitatively evaluated by calculating the PCC between predicted and true expression values. The results demonstrate that R4ST consistently outperforms existing mainstream imputation methods across most cell types (Fig. 2G). In particular, the imputation performance for marker genes of OD Mature and inhibitory cells is especially notable (Fig. 2H). These results indicate that R4ST can capture cell type–specific expression patterns from scRNA-seq data and transfer them to ST data, improving the reliability of cell type identification and functional analysis.

### 3.3 R4ST corrects false-positive expression patterns and improves biological interpretability in ST data analysis

To further validate R4ST’s performance improvement in downstream analyses, we systematically evaluated its biological effectiveness based on key gene and biological interpretability.

**Figure 3:**
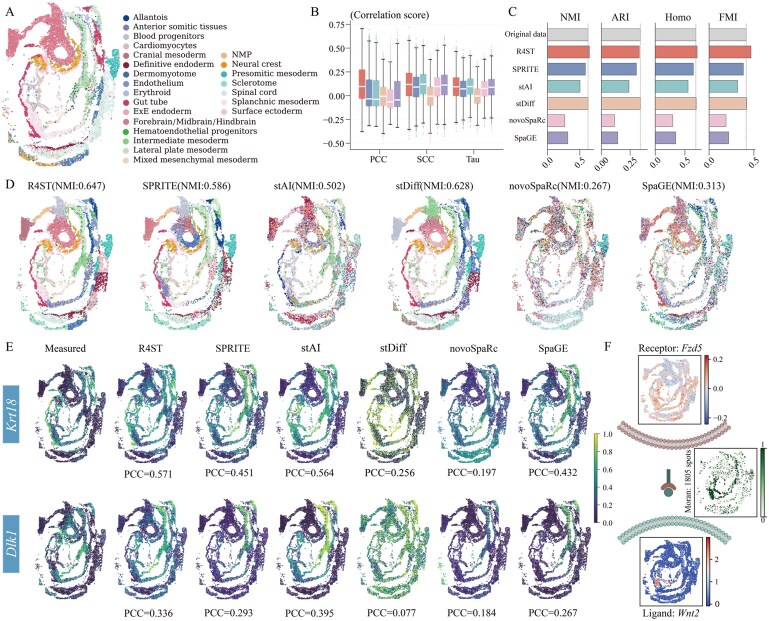
Performance evaluation of R4ST and competing methods on seqFISH mouse gastrulation dataset. (A) Spatial distribution of original cell-type labels. (B) Box plots of coefficients for different methods. (C) Bar plots of clustering consistency for various methods. (D) Spatial visualization of clustering results for R4ST and competing methods, with colors matched to ground-truth labels in (A). (E) Spatial expression patterns of cell-type-specific marker genes in measured versus R4ST-predicted datasets. Values are zero-clipped and [0,1]-normalized. (F) Significant interaction regions of ligand-receptor interaction based on R4ST-imputed data.

First, we validated using a seqFISH dataset from mouse gastrulation (Fig. 3A). This dataset contains 7656 cells but measures only 351 genes. To ensure consistency and fairness of the input across methods, only 250 genes shared between the ST dataset and reference scRNA-seq data were used as input, and expression was predicted for 2295 genes annotated as HVGs in the scRNA-seq data. In prediction performance, R4ST demonstrated a clear advantage across all three metrics (Fig. 3B). Compared with other methods, PCC was improved by up to about 5%. To assess the impact of gene imputation on cellular spatial distribution characterization, cell clustering analysis was performed based on the imputed gene expression matrix. The results show that R4ST consistently outperformed other methods in consistency metrics (Fig. 3C and D), highlighting its superiority in preserving and enhancing cell-type discrimination. Similarly, compared with clustering based directly on the original seqFISH data, using imputed data led to substantial improvement.

Next, to evaluate the generalization capability of R4ST in recovering spatial patterns of genes not involved in model training, we focused on markers with established biological roles. Specifically, we examined *Krt18* and *Dlk1*, which are pivotal in early embryonic development. *Krt18* is involved in maintaining epithelial cell cytoskeletal structure and is an important marker of early epithelial lineage differentiation and tissue morphogenesis (Lim *et al.* 2020, Liang *et al.* 2023). *Dlk1*, as a regulatory factor associated with the Notch signaling pathway, plays an important role in embryonic stem cell differentiation, mesoderm development, and organogenesis (Traustadóttir *et al.* 2019). The results show that for these key genes, the proposed method exhibits higher correlations (Fig. 3E). At the spatial level, R4ST-predicted high-expression regions are more consistent with true spatial patterns, enabling more accurate recovery of their localized enrichment within embryonic tissues. In contrast, other imputation methods show noticeable deviations in high-expression region localization and expression gradient characterization. These results (Fig. 3D and E) underscore a pivotal advantage of R4ST from its unique architecture. While stDiff lacks spatial topological constraints leading to fragmented gene patterns, and stAI incurs an over-smoothing effect blurring cell-type boundaries, R4ST simultaneously optimizes both dimensions by leveraging dual learning channels with distinct semantic roles. The inductive channel captures cell-type-specific expression signatures to ensure accurate recovery of marker gene profiles, while the transductive channel utilizes the full spatial graph to maintain global structural consistency. By anchoring these features within a shared latent space, R4ST effectively bridges the gap between global cellular identities and local cell-type-specific gene expressions. This enables the restoration of biologically faithful patterns, such as those of *Krt18* and *Dlk1*, without compromising underlying cellular identity or introducing out-of-distribution artifacts.

To evaluate R4ST’s ability to resolve spatial biological signals, we further analyzed cell-cell communication (CCC) and applied SpatialDM (Li *et al.* 2023) to detect biologically meaningful ligand–receptor interactions. The results show that the 250 input genes covered 22 ligand–receptor pairs, among which 21 pairs were successfully recovered based on the imputed data from R4ST, with only one interaction not recovered (Table S2). In addition, when R4ST was used to impute all undetected genes, the capacity to identify spatial ligand–receptor interactions was substantially enhanced, resulting in a total of 61 potential spatial interactions (Fig. S3). Taking the representative interaction pair *Wnt2*–*Fzd5* shown in the Fig. 3F as an example, its active regions are distributed across multiple cell types closely related to embryonic development, including the lateral plate mesoderm, the allantois, and the visceral mesoderm. These results are consistent with previous findings that the secretion of *Wnt2* from the lateral plate mesoderm (LPM) and its interaction with the receptor *Fzd5* in the adjacent endoderm are essential for the specification and morphogenesis of foregut-derived organs, such as the liver and lungs (Poulain and Ober 2011), indicating that R4ST not only complements missing gene but also reveals spatial CCC that are difficult to directly observe from the original data.

To further validate the biological interpretability and reliability of R4ST results, we evaluated the model using a STARmap dataset derived from mouse Visual cortex cells. As an image-based ST technology, STARmap relies heavily on probe design during gene detection, and its measurements are susceptible to substantial dropout and background noise. By incorporating scRNA-seq data as a reference, R4ST can effectively learn the underlying gene expression distributions during the prediction process, thereby substantially mitigating the influence of these noises and improving the fidelity of spatial gene expression patterns (Fig. S4). Specifically, in the original STARmap data, the genes *Rspo1* and *Fezf2* exhibit pronounced false-positive spatial signals, whereas after imputation with R4ST, their spatial expression patterns show a high degree of consistency with those reported in the Allen Brain Atlas (Yao *et al.* 2023). Furthermore, for genes not directly measured by the STARmap technology, the spatial expression patterns predicted by R4ST are also in agreement with the corresponding gene expression profiles in the Allen ISH dataset, demonstrating that R4ST possesses strong generalization and extrapolation capabilities for unobserved genes. Differential expression analysis performed on the imputed spatial transcriptomic data identified multiple cell type-specific genes that have been reported in previous studies, such as *Opalin* in oligodendrocytes (Kippert *et al.* 2008) and *Fcgrt* in endothelial cells (Pannek *et al.* 2023). Notably, this biologically relevant information is absent from the original STARmap data.

Taken together, these results demonstrate that R4ST not only improves the completeness of different types of ST data but also substantially enhances their biological interpretability.

### 3.4 R4ST restores fine-grained anatomical architectures and functional landscapes in sequencing-based spatial transcriptomics

**Figure 4:**
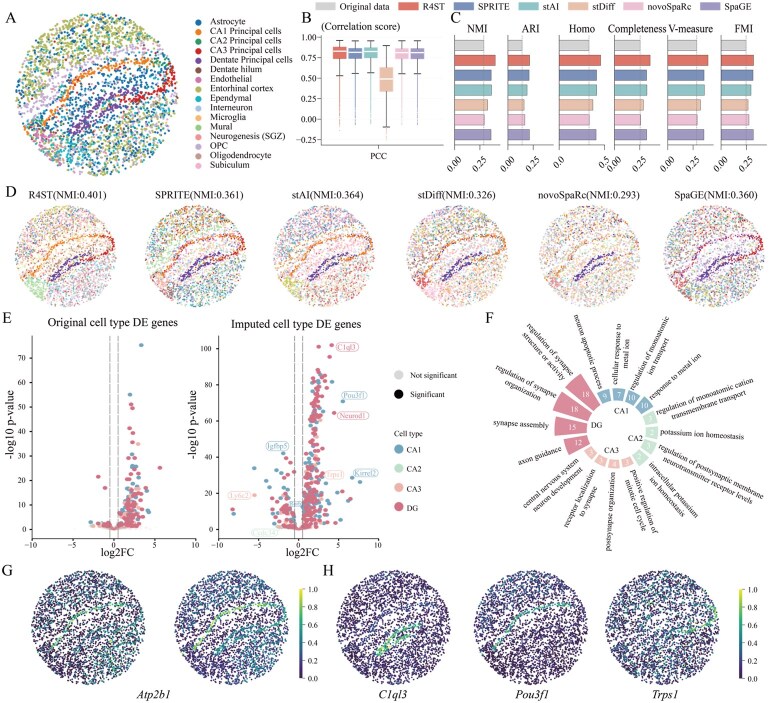
Application of R4ST to Slide_seq V2 data. (A) Spatial distribution of cell-type labels. (B) Box plots for coefficients between different methods. (C) Bar plots of clustering consistency for various methods. (D) Spatial visualization of clustering results for competing methods. (E) DEs for four major cell types identified from original and R4ST-imputed data. (F) GO analysis of cell-type-specific DE genes. Bar heights represent −log_10 (*P*-value) and numbers indicate the count of genes enriched in each GO term. (G) Spatial expression patterns of the CA1 marker gene *Atp2b1* in the original (left) and R4ST-imputed (right) datasets. (H) Spatial visualization of novel cell-type-specific DE genes identified by R4ST.

We also extended our evaluation to sequencing-based data. This type of data inherently suffers from severe dropout issues, causing inaccuracies in downstream analyses. We used a Slide-seq v2 dataset from the mouse hippocampus (Fig. 4A) comprising 6000 cells and 1000 genes. For fair comparison, we restricted input to 950 genes shared between the ST dataset and scRNA-seq reference, then predicted expression for 3000 HVGs identified in the reference data.

To evaluate clustering coherence, we employed cell-type deconvolution and annotated cell labels (Cable *et al.* 2022b; Zhang *et al.* 2025). In sequencing-based spatial data, through gene density is less of a primary constraint compared to capture-based methods, cellular mixing and stochastic dropout remain significant challenges. Benchmarking analysis showed R4ST consistently maintained superior accuracy (Fig. 4B and C). While all imputation methods improved clustering consistency, R4ST achieved the most substantial gains, increasing the UMI-based coherence metrics from 0.289 to 0.401 (Fig. 4C and D). In the mouse hippocampus, the DG, CA1, CA2, and CA3 layers represent critical distinct cell populations. As shown in clustering results (Fig. 4D), R4ST had the highest alignment with anatomical cell labels across these four regions, while alternative methods showed boundary blurring and spatial mixing.

The biological utility of R4ST was further validated through differential expression and GO enrichment analyses. Utilizing C-SIDE (Cable *et al.* 2022a), we identified a broader repertoire of DEs in R4ST-imputed data compared to raw transcripts (Fig. 4D). R4ST not only enabled the discovery of previously obscured transcripts but also refined spatial expression patterns of genes already detected in raw data. By mitigating technical noise and stochastic dropouts, R4ST restored spatial fidelity of key markers. For example, the expression of *Atp2b1*, a known marker for the CA1 pyramidal layer (Stickels *et al.* 2021), had a much sharper and more biologically coherent distribution after imputation, eliminating diffusion artifacts in raw sequencing data.

Moreover, R4ST allowed for precise localization of functionally significant markers. We identified *C1ql3* as highly enriched in the Dentate Gyrus (DG, Fig. 4E). As a member of the C1q complement family, *C1ql3* is a known synaptic organizer essential for recruiting postsynaptic receptors and maintaining excitatory synapses (Yuzaki 2017). This molecular recovery is directly supported by our GO enrichment results (Fig. 4F), showing the DG region is predominantly associated with synapse assembly, regulation of synapse structure or activity, and axon guidance (Cheng *et al.* 2021). Other regions also showed distinct functional specialization in the imputed data. The CA2 region is a hub for a potent excitatory synaptic circuit, linking cortical inputs with hippocampal outputs (Srinivas *et al.* 2017). Our GO analysis for the CA2 region showed unique signatures of potassium ion homeostasis and intracellular potassium ion transport (Fig. 4F). These molecular features are essential for modulating hippocampal network excitability and population activity in vivo, as well as the unique peak-firing properties and excitability of CA2 neurons (Whitebirch *et al.* 2022). The CA3 region showed enrichment in postsynapse organization and positive regulation of the mitotic cell cycle (Fig. 4F), consistent with the extensive molecular requirements for maintaining its complex recurrent collateral network (Sun *et al.* 2017). Finally, spatial visualization confirmed that the CA1 marker *Pou3f1* (Zhong, Ang and Chen 2024) has significantly sharper cell-type specificity, while *Trps1* showed highly specific enrichment within the CA3. These results demonstrate that R4ST not only led to superior spatial clustering coherence that aligns more closely with anatomical structures but also unmasked subtle biological variations, enabling identification of rare cell types and differential expression patterns previously obscured by technical noise.

## 4 Conclusion and discussion

In this study, we presented R4ST, an innovative graph-based framework designed to bridge the gap between spatial granularity and transcriptome coverage in ST data. By leveraging a shared latent space anchor and dual-channel learning, R4ST can accurately recover a large number of HVGs from a small subset of genes, outperforming existing generative and embedding-based methods. Notably, R4ST not only imputes image-based ST data to overcome technical constraints on gene coverage but also effectively mitigates high-ratio dropout in sequencing-based ST data. Across multiple datasets, our results demonstrate that R4ST identifies novel spatial patterns associated with rare cell types, reduces technical noise, and enables more reliable biological interpretation. To ensure optimal performance, practical guidelines for scRNA-seq reference selection and grid-search-based hyperparameter optimization are provided in Supplementary Section 4.

While R4ST demonstrates robust performance on paired datasets, generalizing it to mismatched scRNA-seq and spatial transcriptomics samples poses a challenge, especially with high inter-patient variability or batch effects. Future work will involve integrating pre-trained gene expression foundation models with semantic encoders for clinical metadata. By leveraging representations from large-scale single-cell and spatial datasets, we aim to improve the robustness and transferability of R4ST across diverse tissues. In summary, R4ST offers a powerful framework for converting sparse, noisy spatial data into high-fidelity molecular maps, advancing tissue heterogeneity and spatial biology research.

## Supplementary Material

btag228_Supplementary_Data

## Data Availability

The data underlying this article are available in the Supplementary Materials (Section 3), where relevant links and identifiers are provided.
