# Supplementary material for: R4ST: a reference-guided graph-generative model for robust reconstruction of spatial transcriptomic profiles
Source: Bioinformatics. 2026 Jul 7;42(Suppl 1):btag228. doi: 10.1093/bioinformatics/btag228 (PMC13340175; doi:10.1093/bioinformatics/btag228)
Supplement: btag228_Supplementary_Data [file btag228_supplementary_data.pdf]

Supplementary Materials for

R4ST: A Reference-guided Graph-generative Model for Robust Reconstruction of Spatial Transcriptomic Profiles

Mingyue Wei[1], Wenrui Li[2], Wei Zhang[1,*], Zhi-Ping Liu[1,*]

[1] Department of Biomedical Engineering, School of Control Science and Engineering, Shandong University, Jinan, Shandong 250061, China

[2] Department of Automation, Tsinghua University, Beijing, 100084, China

*To whom correspondence should be addressed. Tel: +86 0531 88364052; Email: zpliu@sdu.edu.cn

Correspondence may also be addressed to Wei Zhang. Email: zw@sdu.edu.cn

Supplementary Notes

1. Detailed Evaluation Framework for Spatial Gene Imputation

1.1 Metrics for Gene Expression Fidelity

These metrics quantify the similarity between the predicted gene expression y' and the ground truth observations y at the single-cell/spot level:

Pearson Correlation Coefficient (PCC): Measures the linear correlation between the imputed and observed expression vectors.

$$PCC = \frac{\sum_{i=1}^{n}(y_i - \bar{y})(y_i' - \bar{y'})}{\sqrt{\sum_{i=1}^{n}(y_i - \bar{y})^2 \sum_{i=1}^{n}(y_i' - \bar{y'})^2}}$$

where n is the number of genes, and $\bar{y}$, $\bar{y'}$ are the mean values.

Spearman's Rank Correlation Coefficient (SCC): Assesses the monotonic relationship by using the rank of expression values, providing robustness against technical outliers.

$$SCC = 1 - \frac{6\sum d_i^2}{n^3 - n}$$

where $d_i = rank(y_i) - rank(y_i')$ is the difference between the ranks of each observation.

Kendall's Tau (Tau): Evaluates the ordinal association and rank consistency, defined as:

$$Tau = \frac{(\text{number of concordant pairs}) - (\text{number of discordant pairs})}{\frac{1}{2}n(n-1)}$$

1.2 Metrics for Clustering Consistency

To evaluate whether the imputed gene expression profiles enhance the preservation of biological structures and spatial domains, we performed UMAP dimension reduction followed by K-means clustering on the imputed data. The resulting clusters were compared against the

ground-truth cell-type annotations C. For all datasets, the number of clusters K in the K-means algorithm was pre-set to match the total number of unique cell types (or spatial domains) identified in the ground-truth annotations. The following metrics were employed to quantify clustering consistency:

Normalized Mutual Information (NMI): NMI measures the similarity between two clustering results by normalizing the mutual information. We utilized the geometric mean normalized mutual information:

$$NMI(C,C') = \frac{MI(C,C')}{\sqrt{H(c)H(C')}}$$

where $MI(C,C')$ represents the mutual information between clusterings C and C', and H(.) denotes the Shannon entropy.

Adjusted Rand Index (ARI): ARI evaluates the similarity between two clusters by considering the number of pairs assigned to the same or different clusters, adjusted for the probability of random labeling:

$$ARI(C,C') = \frac{RI(C,C') - E[RI(C,C')]}{max\big(RI(C,C')\big) - E[RI(C,C')]}$$

The Rand Index (RI) is defined as:

$$RI(C,C') = \frac{a+b}{a+b+c+d}$$

where:

- **a:** Number of pairs assigned to the same cluster in both C and C'.
- **b**: Number of pairs assigned to different clusters in both C and C'.
- **c**: Number of pairs in the same cluster in C but in different clusters in C'.
- **d**: Number of pairs in different clusters in C but in the same cluster in C'.

Homogeneity: Homogeneity measures the extent to which each cluster contains only members of a single actual biological class:

$$h(C|C') = 1 - \frac{H(C|C')}{H(C)}$$

where $H(C|C')$ is the conditional entropy of the classes given the cluster assignments. A cluster is perfectly homogeneous if all its members belong to the same class.

Completeness: Measures the extent to which all members of a given actual biological class are assigned to the same cluster:

$$c(C|C') = 1 - \frac{H(C'|C)}{H(C')}$$

A clustering result is perfectly complete if all elements of a specific class are grouped within the same cluster.

V-measure: The V-measure is the harmonic mean of homogeneity h and completeness c, providing a balanced overall assessment of clustering quality:

$$v(C, C') = \frac{(1+\beta) \cdot h \cdot c}{\beta \cdot h + c}$$

where β is a weight coefficient, typically set to 1.

Fowlkes-Mallows Index (FMI): FMI is the geometric mean of precision and recall, calculated based on the pairs of samples:

$$FMI(C, C') = \sqrt{\frac{TP}{TP+FP} \cdot \frac{TP}{TP+FN}}$$

where:

- **TP (True Positive)**: Pairs that are in the same cluster in both C and C'.
- **FP (False Positive)**: Pairs that are in the same cluster in C but in different clusters in C'.
- **FN (False Negative)**: Pairs that are in different clusters in C but in the same cluster in C'.

1.3 Baseline Methods and Parameter Configurations

To ensure a robust and unbiased benchmark, we compared R4ST with five prevailing

methodological paradigms in the field of spatial transcriptomics. All baseline methods were executed using their official implementations (e.g., Python packages or GitHub repositories) with default parameter settings as recommended by the respective authors to ensure a fair comparison:

SPRITE: A meta-algorithm that refines predictions from existing methods (e.g., SpaGE, Tangram) by integrating gene correlation networks and spatial neighborhood graphs through information propagation.

stAI: An encoder-decoder-based model to jointly embed two datasets and perform the downstream annotation and imputation tasks.

stDiff: A generative approach utilizing latent diffusion models to capture spatial gene expression distributions.

novoSpaRc: An optimal transport-based method that reconstructs spatial gene expression by mapping scRNA-seq to a virtual or physical space.

SpaGE: A statistical method that utilizes singular value decomposition (SVD) to project scRNA-seq and ST data into a shared space.

The specific versions of the software and any necessary environment configurations are detailed in the accompanying code repository.

2. R4ST

2.1 R4ST Architecture

To capture both localized spatial context and global expression patterns, R4ST employs a dual-channel learning strategy for feature integration. The process is articulated as follows:

Graph inductive learning channel: This module employs a two-layer GraphSAGE. The input layer dimension corresponds to the number of shared genes between the ST and scRNA_seq. The first GraphSAGE layer projects features into a 512-dimensional hidden space, and the second layer outputs a 1024-dimensional node feature vector $\boldsymbol{Z}_1$.

Graph transductive learning channel: A Variational Graph Autoencoder (VGAE) is used to extract global transcriptomic patterns. The input layer (matching the number of shared genes)

is first processed by a shared Graph Convolutional Network (GCN) layer, which projects the data into a 512-dimensional hidden space to capture common spatial-transcriptomic features. Following the shared layer, the network branches into two parallel GCN layers to deriving the mean μ, and the variance σ of the 1024-dimensional latent representation $\boldsymbol{Z}_2$.

Feature Integration and Latent Representation: The node features from the Graph inductive learning channel $\boldsymbol{Z}_1$ are then fused with the latent representation $\boldsymbol{Z}_2$ through the Graph transductive learning channel, generating a comprehensive 1024-dimensional latent embedding.

Decoder and Imputation Output: The fused latent embedding is passed through a final fully connected (FC) layer, which decodes the representation into the reconstructed space of all target genes. This process enables the high-fidelity imputation of missing gene expression data by leveraging the synergistic information from both the spatial and transcriptomic modalities.

2.2 Hyperparameters and training strategy

To evaluate the robustness and generalization capability of R4ST, we maintained highly consistent hyperparameter configurations across all benchmarking datasets. This approach ensures that the observed performance gains are inherent to the model architecture rather than the result of dataset-specific fine-tuning.

The core architecture of the model, specifically the number and dimensionality of the hidden layers described in the previous section, remained identical for all experiments. For all datasets, each spatial spot was embedded into a 1024-dimensional latent vector. To accommodate different spatial transcriptomics platforms, we only adjusted the input and output layer dimensions to match the specific number of shared and target genes for each dataset, while keeping the internal feature extraction modules constant. This consistency highlights R4ST's ability to handle diverse technologies—varying in feature distributions and noise levels—without the need for meticulous manual adjustments.

The model optimization is governed by a multi-task objective function:

$$Loss_{total} = loss_{feature} + \alpha \cdot loss_{graph} + \beta \cdot loss_{kl}$$

where $loss_{feature}$ denotes the training feature loss, $loss_{graph}$ denotes the graph reconstruction loss, and $loss_{kl}$ represents the KL divergence for variational regularization. Given that the model aims to predict thousands of missing genes from a limited set of shared features, it requires maximum focus on high-dimensional feature synthesis. Also, through empirical observation, we found that prioritizing feature reconstruction accuracy is critical for the recovery of missing gene expression values. Excessive regularization from global spatial topology or latent distribution constraints can potentially lead to over-smoothing in high-dimensional gene space. Consequently, we set the default weights α and β to near-zero for all benchmarking experiments to maximize the model's focus on fine-grained feature imputation via $loss_{feature}$.

Moreover, the values in $\boldsymbol{Y}_{st\_test}$ and $\boldsymbol{Y}_{sc\_test}$ are compared with the corresponding predictions to compute the spatial loss (st_{loss}) and single-cell loss (st_{loss}), respectively, which are used to evaluate the model:

$$st_{loss} = MSE\left(\boldsymbol{Y}_{st_{test}}, \boldsymbol{Y}'\right),$$

$$st_{loss} = MSE\left(\boldsymbol{Y}_{sc_{test}}, \boldsymbol{Y}'\right).$$

We employed a standardized training protocol to ensure a fair comparison across all scenarios. The model was optimized using the Adam optimizer with a fixed learning rate of 0.00001. To accommodate the varying sample sizes and convergence rates of different tissue types, an Early Stopping mechanism was implemented. Specifically, the training process was monitored based on the spatial loss st_{loss} calculated on a held-out validation set. Since st_{loss} directly reflects the model's accuracy in imputing spatial gene expression, it serves as the most reliable indicator of generalization performance. The training was terminated if the validation st_{loss} failed to improve for a predefined number of epochs (patience). While the learning rate and architecture were static, the patience for early stopping was adaptively set based on the dataset scale. This approach ensures that the model achieves optimal convergence on each specific dataset while maintaining a unified training framework.

2.3 Sensitivity analysis of hyperparameters α and β

To evaluate the robustness of our model, we performed a grid search for hyperparameters α and β across three diverse datasets (MERFISH_MOP, MERFISH_Hypothalamic, and seqFISH). For each configuration, we fixed the training process at 200 epochs to ensure comparability across trials. We varied both parameters across the range $\{0.0,0.5,1.0\}$ and measured the model's performance based on the median PCC metric.

The sensitivity analysis results are illustrated in Supplementary Figure S5. The observed performance fluctuations across the grid confirm that while model accuracy is influenced by the balancing of α and β, the results remain within a reasonably stable range across different biological contexts. Specifically:

Dataset-Specific Optimal Points: We observe that the optimal configuration varies by dataset. For instance, $(\alpha = 0.5, \beta = 0.5)$ yields the best performance for the MERFISH_MOP dataset, whereas $(\alpha = 0.0, \beta = 1.0)$ is optimal for seqFISH. This variance underscores the necessity of dataset-specific tuning to maximize model performance.

Parameter Stability: The heatmaps show that performance gradients are relatively smooth, suggesting that the model is not overly sensitive to minor perturbations, which ensures reliable performance for general use cases.

Default Parameter Recommendation: While dataset-specific tuning is recommended for optimal results, we propose default hyperparameters of $\alpha = 0.001$ and $\beta = 0.1$ for general applications. Based on our sensitivity analysis, these values consistently provide a robust balance across diverse spatial transcriptomics platforms, mitigating the risk of performance instability (e.g., negative PCC) observed under more extreme parameter settings.

2.4 Platform

The model was trained and tested on a high-performance workstation (Dell Precision 7960 Tower) running Ubuntu 22.04 LTS, equipped with an NVIDIA RTX A6000 GPU (48GB GDDR6 VRAM). The substantial VRAM capacity allowed for efficient processing of large-

scale spatial transcriptomics datasets with minimal computational latency.

3. Datasets and Data processing

3.1 Datasets

To evaluate the capability of R4ST in spatial gene imputation, we performed comprehensive comparisons and analyses using spatial transcriptomics (ST) data generated from multiple leading technologies, including MERFISH, STARmap, seqFISH, and Slide-seq v2. For each ST dataset, a corresponding scRNA-seq dataset from the same tissue and species was utilized as a reference for the imputation task. All spatial and scRNA-seq datasets used in this study were obtained as pre-processed count matrices from a previously published benchmarking study[1].

(1) **Mouse Primary Motor Cortex (MERFISH)**: This dataset was generated using MERFISH technology, comprising 6,963 spots and 254 spatially resolved genes, accessible at Dataset1_spatial[2]. It is paired with a scRNA-seq reference containing 25,186 cells and 7,240 genes, accessible at Dataset1_scRNA[2].

(2) **Mouse Hypothalamic Preoptic Region (MERFISH)**: Another MERFISH dataset focusing on the hypothalamic preoptic region, containing 4,975 spots and 154 genes, found at Dataset2_spatial[3]. The associated scRNA-seq reference consists of 18,646 cells and 31,299 genes, found at Dataset2_scRNA[3].

(3) **Mouse Visual Cortex (STARmap)**: This dataset was acquired via STARmap technology, encompassing 1,549 spots and 1,020 genes within the mouse visual cortex, available at Dataset3_spatial[4]. It is integrated with a scRNA-seq reference of 34,043 cells and 14,249 genes, available at Dataset3_scRNA[5].

(4) **Mouse Gastrulation (seqFISH)**: Generated using seqFISH technology, this dataset captures 8,425 spots and 351 genes during mouse organogenesis, accessed at Dataset4_spatial[6]. It is complemented by a scRNA-seq atlas of mouse gastrulation containing 19,103 cells and 4,651 genes, accessed at Dataset4_scRNA[6].

(5) **Mouse Hippocampus (Slide-seq v2)**: This dataset utilizes Slide-seq v2 technology, offering near-cellular resolution with 6,000 spots and a full-transcriptome coverage of 23,264 genes, accessed at Dataset5_spatial[7]. A reference scRNA-seq dataset of 22,095 cells and 10,000 genes from the adult mouse brain was used for imputation, accessed at Dataset5_scRNA[8].

For more detailed information, please see Supplementary Table S1.

3.2 Data processing

To ensure the quality and biological relevance of the input data, we performed a series of preprocessing steps using the Scanpy package[9]. The workflow includes gene and cell filtering followed by normalization.

Quality Control and Filtering: We employed the Scanpy library for data quality control. For scRNA-seq datasets, genes with an average expression value below 0.01 were excluded to ensure the robustness of the features. However, for datasets with limited overlapping features, gene filtering was bypassed to maximize the retention of shared information between spatial and single-cell modalities. Additionally, for large-scale datasets (e.g., Dataset 2_MERFISH), we removed cells with fewer than 7,000 total counts to reduce computational complexity and focus on high-quality cellular profiles.

Normalization and Transformation: After filtering, the raw count matrices for both spatial transcriptomics and scRNA-seq were normalized by scaling the total counts per cell/spot to a fixed target sum. To stabilize variance and mitigate the impact of outliers, the normalized data were then subjected to a log(1+x) transformation. This standardized preprocessing pipeline ensures that the input features are directly comparable across different experimental batches and biological conditions.

3.3 Selection of Cell Type-Specific Marker Genes

To evaluate the biological fidelity of the imputed data, we identified representative marker genes for each cell type using the Scanpy framework. We employed the rank_genes_groups function with the Wilcoxon rank-sum test to perform differential expression analysis across

distinct cell clusters. For each identified cell class, genes were ranked based on their statistical scores. The top 10 genes for each category were then extracted as the definitive marker set. These genes served as the ground truth for calculating the Pearson Correlation Coefficient (PCC) between predicted and true expression levels, as shown in Figure 2G.

4. Best practices

To maximize the performance and reliability of R4ST, we provide the following best practices for reference selection and model configuration:

1. **Reference Dataset Requirements**

(1) Biological Representation: The scRNA-seq reference should ideally include all primary cell-type lineages identified in the target tissue. Missing major cell populations can lead to biased imputation in spatial regions where those cells reside.

(2) Sample Source: For best results, the scRNA-seq reference and ST data should ideally originate from the same species and the same tissue type to minimize biological batch effects and ensure accurate cell-state mapping.

2. **Hyperparameter Optimization**

The model's performance is sensitive to the weighting between $loss_{graph}$, and $loss_{kl}$. We recommend a grid search approach:

(1) Loss Weighting: The model's performance relies on the balance between $loss_{feature}$, $loss_{graph}$, and $loss_{kl}$. Given that these components operate on different scales, we recommend performing a grid search on the weighting hyperparameters α and β (suggested range: $[0,1]$). The goal is to identify a configuration where $loss_{graph}$ and $loss_{kl}$ effectively preserves spatial topology without overpowering the $loss_{feature}$ required for high-fidelity gene expression reconstruction.

(2) Patience: For training, we advise setting the patience for early stopping adaptively based on the dataset scale. Specifically, larger datasets often require an increased patience value to allow the model sufficient iterations to fully capture complex gene expression features and

converge stably.

3. **Computational Considerations Memory Management**

Maintaining an appropriate ratio between the number of spatial spots and reference cells is critical for robust neighborhood graph construction and computational efficiency. An excessive reference size relative to spatial spots can lead to a sparse or noisy neighborhood graph, significantly increasing the computational overhead during $loss_{graph}$ calculation. For datasets exceeding 50,000 cells, we recommend sub-sampling the reference to a representative subset, ensuring that the reference density adequately captures the diversity of the spatial sample without overwhelming the graph-based topology.

5. Reference

[1] Li B, Zhang W, Guo C, et al. Benchmarking spatial and single-cell transcriptomics integration methods for transcript distribution prediction and cell type deconvolution[J]. Nature Methods, 2022, 19(6): 662-670.

[2] Booeshaghi A S, Yao Z, van Velthoven C, et al. Isoform cell-type specificity in the mouse primary motor cortex[J]. Nature, 2021, 598(7879): 195-199.

[3] Moffitt J R, Bambah-Mukku D, Eichhorn S W, et al. Molecular, spatial, and functional single-cell profiling of the hypothalamic preoptic region[J]. Science, 2018, 362(6416): eaau5324.

[4] Wang X, Allen W E, Wright M A, et al. Three-dimensional intact-tissue sequencing of single-cell transcriptional states[J]. Science, 2018, 361(6400): eaat5691.

[5] Tasic B, Yao Z, Graybuck L T, et al. Shared and distinct transcriptomic cell types across neocortical areas[J]. Nature, 2018, 563(7729): 72-78.

[6] Lohoff T, Ghazanfar S, Missarova A, et al. Integration of spatial and single-cell transcriptomic data elucidates mouse organogenesis[J]. Nature Biotechnology, 2022, 40(1): 74-85.

[7] Stickels R R, Murray E, Kumar P, et al. Highly sensitive spatial transcriptomics at near-cellular resolution with Slide-seqV2[J]. Nature Biotechnology, 2021, 39(3): 313-319.

[8] Saunders A, Macosko E Z, Wysoker A, et al. Molecular Diversity and Specializations among the Cells of the Adult Mouse Brain[J]. Cell, 2018, 174(4): 1015-1030.e16.

[9] Wolf F A, Angerer P, Theis F J. SCANPY: large-scale single-cell gene expression data analysis[J]. Genome Biology, 2018, 19(1): 15.

Supplementary Tables

Supplementary Table S1. Summary of the datasets used in this study.

Platform	Tissue	ST		scRNA-seq		Common genes	Input features
		#Spots	#Genes	#Cells	#Genes		
MERFISH	Primary motor cortex	6963	254	7240	25186	252	200
MERFISH	Hypothalamic preoptic region	4975	154	31299	18646	154	110
STARmap	Visual cortex	1549	1020	14249	34041	973	923
seqFISH	Gastrulation	8425	351	4651	19103	295	250
Slide_seq V2	hippocampus	6000	23264	10000	22095	1000	950

Supplementary Table S2. Comparison of measured and R4ST-recovered ligand–receptor pairs.

**Ligand–receptor pairs measured**	**Ligand–receptor pairs recovered by R4ST**
ESAM_ESAM	ESAM_ESAM
CDH2_CDH2	CDH2_CDH2
CDH1_CDH1	CDH1_CDH1
CDH5_CDH5	CDH5_CDH5
PECAM1_PECAM1	PECAM1_PECAM1
DLL3_NOTCH1	DLL3_NOTCH1
**DLL1_NOTCH1**	
COL4A1_ITGA3_ITGB1	COL4A1_ITGA3_ITGB1
COL1A1_ITGA3_ITGB1	COL1A1_ITGA3_ITGB1
WNT3_FZD2_LRP6	WNT3_FZD2_LRP6
WNT5B_FZD2	WNT5B_FZD2
BMP7_BMPR1B_ACVR2A	BMP7_BMPR1B_ACVR2A
BMP7_BMPR1A_ACVR2A	BMP7_BMPR1A_ACVR2A
NODAL_ACVR1C_ACVR2A	NODAL_ACVR1C_ACVR2A
NODAL_ACVR1B_ACVR2A	NODAL_ACVR1B_ACVR2A
BMP2_BMPR1B_ACVR2A	BMP2_BMPR1B_ACVR2A
BMP2_BMPR1A_ACVR2A	BMP2_BMPR1A_ACVR2A
WNT3_FZD2_LRP5	WNT3_FZD2_LRP5
FGF15_FGFR3	FGF15_FGFR3
FGF15_FGFR2	FGF15_FGFR2
FGF17_FGFR4	FGF17_FGFR4
FGF10_FGFR2	FGF10_FGFR2

Supplementary Table S3. 3.1 Benchmark Evaluation of Gene Expression Imputation

(**Dataset 1**: MERFISH_Primary motor cortex; **Dataset 2**: MERFISH_Hypothalamic Preoptic Region; **Dataset 3**: STARmap_Visual Cortex; **Dataset 4**: seqFISH_Gastrulation; **Dataset 5**: Slide_seq V2_ Hippocampus;)

	Method	PCC (Median)	SCC (Median)	Tau (Median)	NMI	ARI	Homo	Completeness	V_measure	FMI
1	R4ST	**0.242**	**0.237**	**0.163**	**0.809**	**0.621**	**0.845**	**0.775**	**0.808**	**0.655**
	SPRITE	0.190	0.075	0.039	0.786	0.608	0.824	0.750	0.785	0.646
	stAI	0.241	0.063	0.021	0.651	0.410	0.687	0.617	0.650	0.458
	stDiff	0.064	0.071	0.048	0.780	0.589	0.820	0.742	0.779	0.629
	novoSpaRc	0.171	0.019	-0.009	0.544	0.331	0.577	0.513	0.543	0.382
	SpaGE	0.186	0.054	0.020	0.581	0.358	0.616	0.547	0.580	0.408
2	R4ST	**0.187**	**0.182**	**0.139**	**0.614**	0.332	**0.707**	0.533	**0.608**	0.445
	SPRITE	0.130	0.119	0.090	0.595	0.338	0.694	0.518	0.589	0.454
	stAI	0.120	0.127	0.097	0.439	0.251	0.506	0.382	0.435	0.369
	stDiff	0.011	0.010	0.007	0.607	**0.357**	0.689	**0.535**	0.602	**0.470**
	novoSpaRc	0.097	0.143	0.110	0.433	0.261	0.497	0.376	0.428	0.379
	SpaGE	0.129	0.149	0.115	0.411	0.203	0.476	0.354	0.406	0.322
3	R4ST	0.162	0.160	0.128	**0.450**	**0.285**	**0.480**	**0.422**	**0.449**	**0.355**
	SPRITE	0.143	0.151	0.121	0.329	0.191	0.352	0.308	0.329	0.266
	stAI	0.151	0.145	0.116	0.327	0.165	0.350	0.267	0.327	0.242
	stDiff	0.061	0.061	0.050	0.283	0.158	0.302	0.306	0.283	0.236
	novoSpaRc	**0.173**	**0.176**	**0.142**	0.305	0.172	0.325	0.308	0.304	0.249
	SpaGE	0.139	0.148	0.119	0.377	0.212	0.401	0.355	0.377	0.287
4	R4ST	**0.038**	**0.141**	**0.111**	**0.647**	0.330	**0.705**	**0.594**	**0.645**	**0.468**
	SPRITE	-0.034	0.091	0.071	0.586	0.304	0.642	0.534	0.583	0.383
	stAI	-0.039	0.120	0.096	0.502	0.240	0.553	0.456	0.500	0.318
	stDiff	-0.007	-0.005	-0.004	0.628	**0.339**	0.690	0.572	0.626	0.418
	novoSpaRc	-0.067	0.096	0.076	0.267	0.115	0.295	0.242	0.266	0.190
	SpaGE	-0.045	0.111	0.089	0.313	0.142	0.345	0.284	0.312	0.218
5	R4ST	**0.829**	0.575	0.474	**0.401**	**0.214**	**0.457**	**0.352**	**0.398**	**0.321**
	SPRITE	0.815	0.592	0.489	0.362	0.209	0.412	0.317	0.359	0.316
	stAI	0.825	0.603	0.509	0.364	0.189	0.414	0.320	0.361	0.296
	stDiff	0.490	0.392	0.315	0.326	0.160	0.372	0.285	0.323	0.267
	novoSpaRc	0.759	0.576	0.473	0.293	0.168	0.331	0.258	0.290	0.275
	SpaGE	0.811	**0.604**	**0.518**	0.360	0.212	0.407	0.318	0.357	0.319

Supplementary Figures

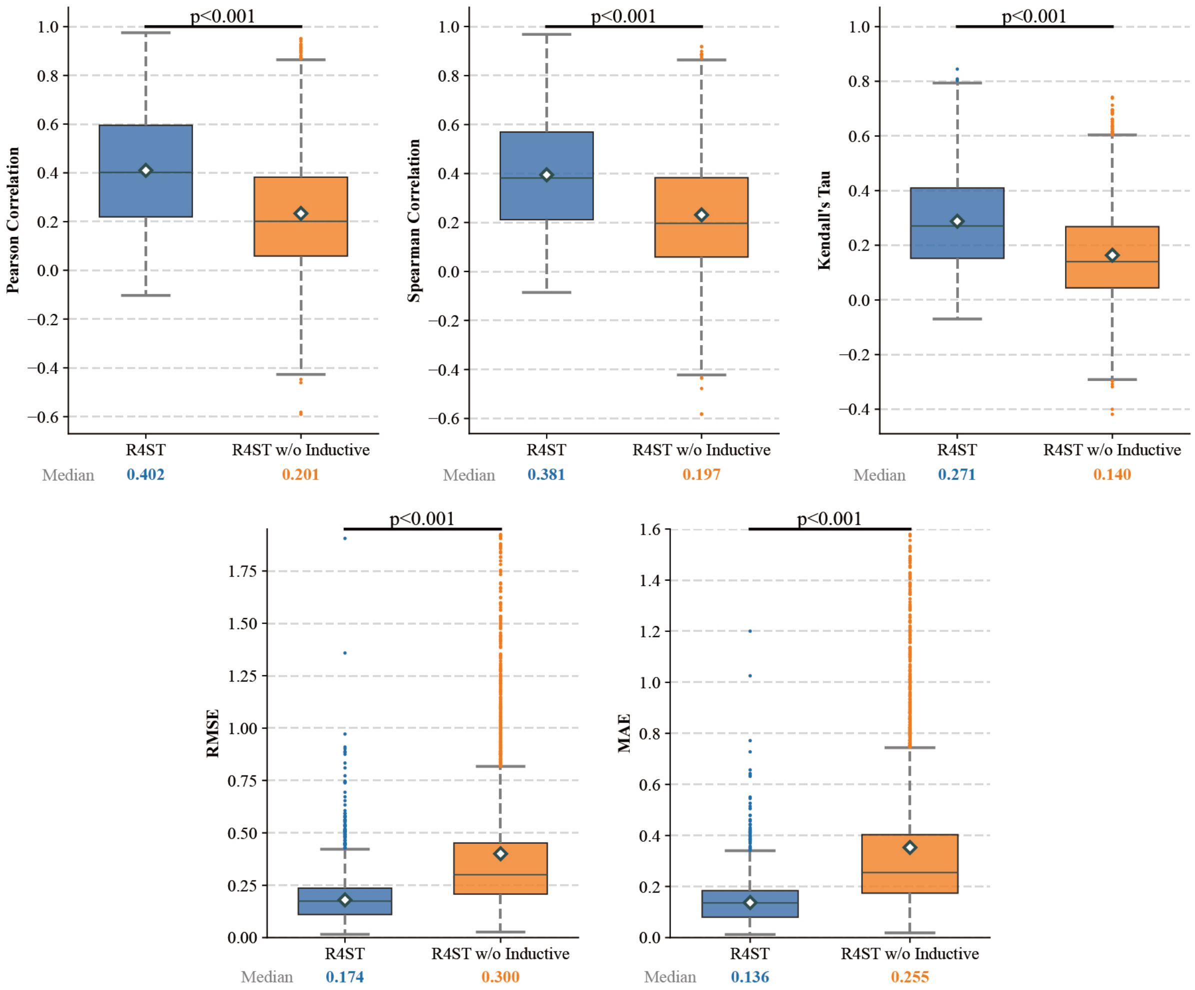

Figure S1. Ablation study of the inductive learning module. Box plots comparing the imputation performance between the full R4ST model (blue) and the model without inductive learning (yellow). The full model consistently outperforms the baseline across all metrics, including correlation coefficients (PCC, SCC, Tau) and error estimates (RMSE, MAE). These results confirm that the GraphSAGE-based inductive channel is essential for capturing transferable spatial patterns and improving predictive accuracy. Statistical significance was determined by the two-sided Wilcoxon rank-sum test ($p < 0.001$).

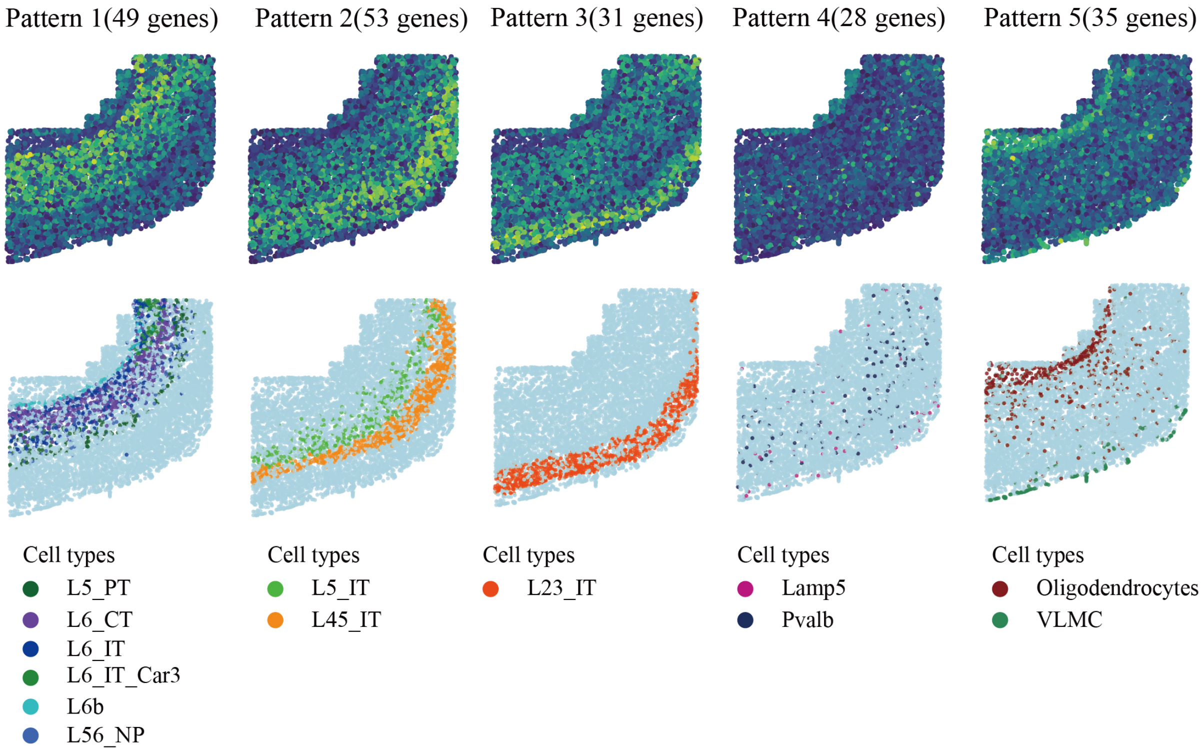

Figure S2. Known spatial patterns in the mouse primary motor cortex (MOp) MERFISH dataset. The number of genes within each identified spatial pattern is indicated. The first row displays the averaged expression levels of measured genes for each pattern, while the second row shows the corresponding co-localized cell types.

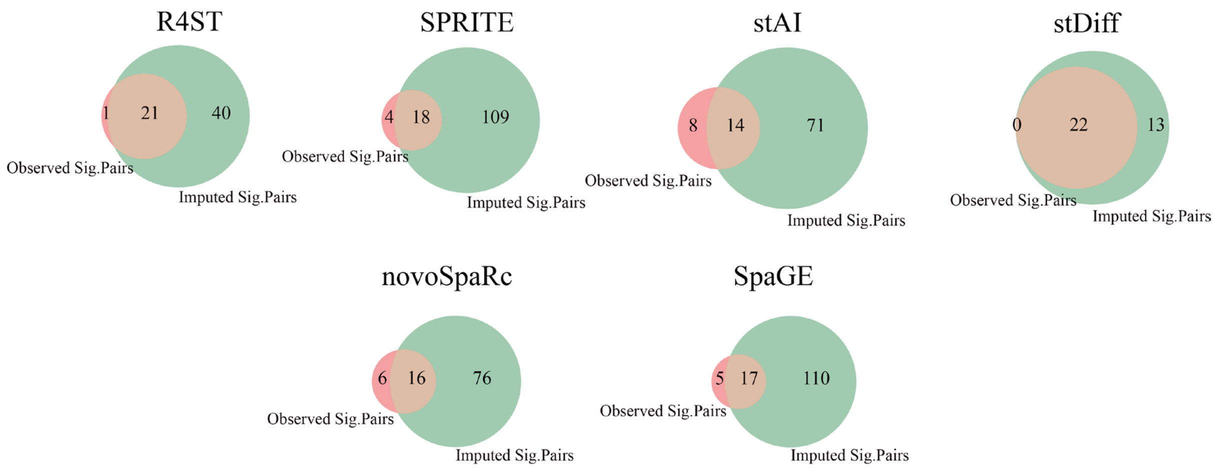

Figure S3. Comparison of ligand-receptor (L-R) pair identification across imputation methods. This figure evaluates the ability of various methods to preserve observed signals (red) and discover novel interactions (green) after imputing 2000 highly variable genes. R4ST demonstrates superior performance by recovering 95.5% (21/22) of observed L-R pairs while uncovering 40 additional significant interactions not detectable in the raw data. Compared to other methods, R4ST provides an optimal balance between signal fidelity and discovery power, as methods like stAI and novoSpaRc identified more novel pairs but at the cost of losing a significant portion of original biological signals.

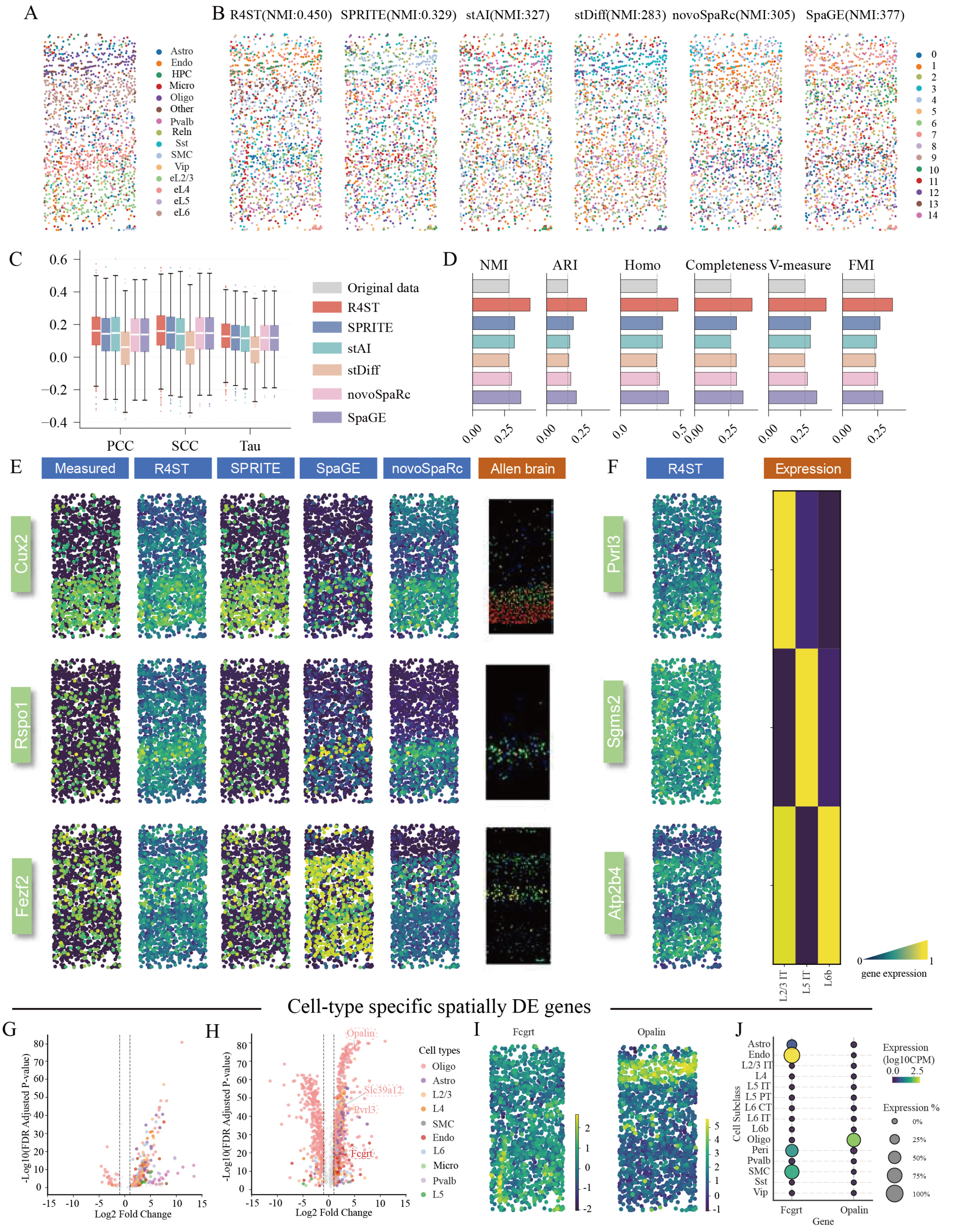

Figure S4. Comprehensive evaluation of R4ST on the STARmap mouse Visual cortex dataset. (A) Spatial distribution of original cell-type labels. (B) Spatial visualization of clustering results for R4ST and competing methods. (C) Box plots for coefficients between different methods. (D) Bar plots of clustering consistency for various methods. (E) Spatial imputation of measured genes. Comparison of measured spatial expression (column 1) against imputed patterns by R4ST and baseline methods(column 2-5) The rightmost column provides

reference In Situ Hybridization (ISH) images from the Allen Brain Atlas for biological validation. (F) Imputation of unmeasured signatures. Spatial prediction of genes absent from the STARmap panel (left). The heatmaps (right) display expression signatures from the snRNA-seq reference, validating the cell-type specificity of R4ST's predictions. (G-H) Identification of cell_type specific spatially DE genes. Volcano plots representing differentially expressed genes (DEGs) identified from (G) measured data and (H) R4ST-augmented data, demonstrating enhanced statistical power after imputation. (I) Prediction of unmeasured markers. Spatial expression patterns for key cell-type marker genes not originally captured in the STARmap experiment.(J) Reference expression profile. Dot plot illustrating the expression intensity and cell-type specificity of marker genes within the snRNA-seq reference dataset.

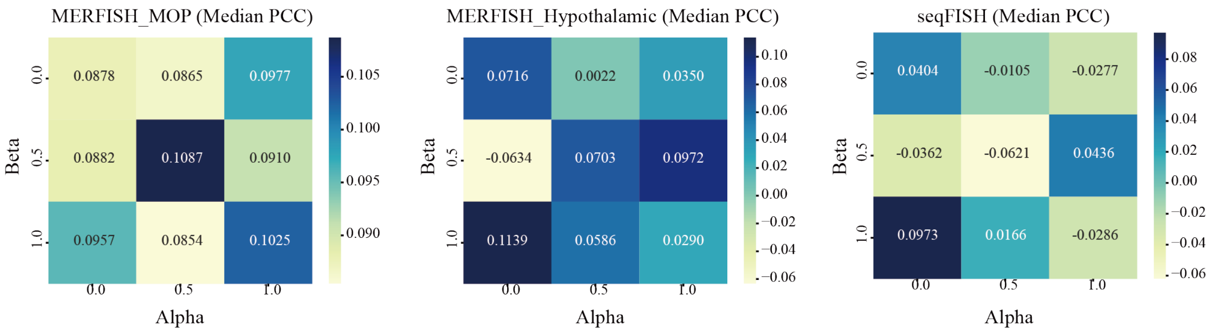

Figure S5. Sensitivity analysis of hyperparameters α and β. The heatmaps display the model performance (median PCC) across different combinations of α and β for three spatial transcriptomics datasets. Darker blue indicates higher performance.
